# Healthcare provider attitudes towards the problem list in an electronic health record: a mixed-methods qualitative study

**DOI:** 10.1186/1472-6947-12-127

**Published:** 2012-11-11

**Authors:** Casey Holmes, Michael Brown, Daniel St Hilaire, Adam Wright

**Affiliations:** 1 Brigham and Women’s Hospital, 1 Brigham Circle, Boston, MA, 02120, USA; 2 Harvard Medical School, Boston, MA, USA; 3 Partners Healthcare, Boston, MA, USA; 4 Harvard University, Health Services, 75 Mt. Auburn St, Cambridge, MA, 02138, USA; 5 Division of General Internal Medicine and Primary Care, Brigham and Women’s Hospital, Boston, MA, USA

**Keywords:** Problem list, Problems, Electronic health record, Standardization, Provider attitudes, EHR

## Abstract

**Background:**

The problem list is a key part of the electronic health record (EHR) that allows practitioners to see a patient’s diagnoses and health issues. Yet, as the content of the problem list largely represents the subjective decisions of those who edit it, patients’ problem lists are often unreliable when shared across practitioners. The lack of standards for how the problem list is compiled in the EHR limits its effectiveness in improving patient care, particularly as a resource for clinical decision support and population management tools. The purpose of this study is to discover practitioner opinions towards the problem list and the logic behind their decisions during clinical situations.

**Materials and methods:**

An observational cross-sectional study was conducted at two major Boston teaching hospitals. Practitioners’ opinions about the problem list were collected through both in-person interviews and an online questionnaire. Questions were framed using vignettes of clinical scenarios asking practitioners about their preferred actions towards the problem list.

**Results:**

These data confirmed prior research that practitioners differ in their opinions over managing the problem list, but in most responses to a questionnaire, there was a common approach among the relative majority of respondents. Further, basic demographic characteristics of providers (age, medical experience, etc.) did not appear to strongly affect attitudes towards the problem list.

**Conclusion:**

The results supported the premise that policies and EHR tools are needed to bring about a common approach. Further, the findings helped identify what issues might benefit the most from a defined policy and the level of restriction a problem list policy should place on the addition of different types of information.

## Introduction

The problem list is a key part of the medical record. At a high level, it lists the patient’s most important health problems and gives the practitioner key information to determine the best treatment plan. Good problem lists are known to improve patient care [1,2] and are used as a resource for clinical decision support tools to prevent medical error [3,4]. Yet, there are issues with problem lists that limit their effectiveness. The three greatest are thought to be:

● No common approach: Practitioners differ in opinion on what should and should not go on the problem list [5,6].

● Completeness (false negatives): major problems are never listed on the patient’s problem list [7].

● Clutter (false positives): Minor or inactive problems accumulate on a problem list making the document unfocused and incomprehensible [8].

With the passage of the HITECH act, most of the medical community is now focused on adopting electronic health records that can improve patient care. As problem lists are more readily shared across providers, the above deficits have become more prominent. Yet, to solve these issues the medical community needs to know more about how practitioners are currently approaching the problem list and the logic behind those decisions. This research is meant to provide such guidance and through this knowledge, encourage the development of a common approach.

## Background

Lawrence Weed created the problem list in the 1960s as part of his recommendations for a problem-oriented medical record [9]. A simple idea, the problem list soon became a commonly accepted part of the paper medical record and is now used in EHRs as well. The American Health Information Management Association (AHIMA) defines the problem list as “a compilation of clinically relevant physical and diagnostic concerns, procedures, and psychosocial and cultural issues that may affect the health status and care of patients” [8]. At its core the problem list is meant to include the most important factors about a patient (largely chronic diseases such as diabetes and chronic heart disease) to allow practitioners to gain a quick sense of the patient and ensure that significant issues that affect treatment decisions are not hidden within the medical note.

Studies show that high quality problem lists directly link to better compliance with best practices in medicine. Hartung et al. found that patients with known systolic dysfunction who had heart failure listed in their problem list were more likely to be prescribed the appropriate drug therapy than those without [1]. In a study looking at encounter notes across consecutive medical visits at six medical clinics, Simborg et al. found that those practitioners who listed the diagnosed problem on the problem list were more likely to follow-up on the issue than those who did not list the diagnosed problem [2].

Practitioner’s ability to quickly appreciate the most important facts about their patients’ impacts their ability to provide high quality healthcare. Therefore, when problems are left out or hidden within a long and cluttered list, the problem lists’ effectiveness is compromised. In order to improve patient care and reap further benefit from the problem list as a data resource, the medical community needs to create clear, consistent, complete, and accurate problem lists. Unfortunately, the medical community’s current approach to the problem list makes inconsistency and error the standard.

### The inconsistencies

In ethnographic and qualitative studies of healthcare providers, Wright et al. found that healthcare providers’ use of the problem list is incomplete, and that attitudes vary across care providers [6,10,11]. Practitioners have developed their own style on how to manage and organize the problem list [5,6]. For example, practitioners may argue that listing a family history of breast cancer directly on the problem list is important for prompting more frequent testing, another practitioner can debate that its inclusion duplicates the family history section and clutters the list. Zhou et al. published similar findings identifying the ambiguity surrounding the definition, use, and benefits of the problem list among different clinician groups, and highlighting the challenges of improving documentation in complex, longitudinal cooperative clinical practices [12].

While these differences are likely frequent, they are problematic for a healthcare system where multiple practitioners are building a patient’s medical record together through the EHR. With no common guidelines for how to approach the problem list, issues such as missing problems [1,2] and lengthiness [8] decrease the potential benefits problem lists could bring to patient care.

### Why address the issue now?

The nationwide transition to the EHR in the United States [13] brings the possibility to standardize parts of the medical record in order to improve patient care. To comply with meaningful use, practitioners must maintain an up-to-date problem list of current and active diagnoses based on ICD-9-CM or SNOMED CT, clinical coding standards designed to classify diseases, symptoms, and other relevant factors about a patient. In addition, at least 80 percent of all unique patients must have at least one entry or an indication of none recorded as structured data in the problem list [14].

Meaningful use standards are preparing the EHR to enable clinical decision support and population management tools [13,14]. These tools depend on reliable platforms of aggregated data such as the medication list [4]. Yet, the problem list is not currently supportive enough for these tools due to its inconsistencies, specifically missing problems [7] and clutter [8]. The problem list will need more than a common language platform to support these tools [10]. If the problem list were standardized – (i.e. policies and tools were designed to ensure that a patient’s problem list was the same regardless of the practitioner(s) who created it) - it could mean improvements to patient care, such as:

● Make it more likely that practitioners identify all the important factors about a patient to determine their best treatment plan [1,2].

● Help to prevent medical errors through clinical decision support tools [3,4].

● Allow for the accurate identification of disease specific populations for quality improvement programs, practitioner report cards, and potential research study participants [10,11].

Further, the problem list is becoming part of the shared medical record across providers and organizations. Specifically, as part of the menu set of meaningful use measures, providers must provide a summary of care record for transitions of care and referrals which must include the problem list [14]. As a likely seed for common shared medical record, creating a common approach to the problem list will be important to reaping the most value from health information exchanges.

### Current policies lack direct guidance for practitioners

Policies on the problem lists can be found through a range of organizations such as AHIMA which released best practices for problem lists in 2008 [8]. Other organizations with policies related to the problem list include Health Level 7 [15] and The Joint Commission [16]. Of course, the federal government also included new requirements for the problem list within the meaningful use standards. The impact of these policies has not yet been measured and, with the exception of meaningful use, it is not clear that any of the policies have been adopted widely.

Based on a review of these current policies, most address what administrators should provide for the problem list with the strongest focus on coding. Guidance for how practitioners should approach, manage, and organize the problem list is largely limited to high level definitions about the problem list. From the policy perspective, practitioners are left to their own personal judgment for what to include and not include in the problem list.

Education and training within healthcare organizations does not appear to provide any further guidance for most practitioners. According to Wright et al. education and training towards the problem list among practitioners interviewed was insubstantial, typically informal, and highly variable [6]. Some healthcare organizations in the United States created their own policies towards the problem list, but it is unclear how effective they are at producing valuable problem lists nor are they in widespread adoption across the United States. Therefore, while policies offer high-level rules, specific guidance to the practitioner on how to construct and maintain an accurate problem list is noticeably absent, leaving room for errors and variation in practice.

Policies and EHR tools are likely the best approach to solving the issues with current electronic problem list [6]. Yet, very little research exists on how practitioners make decisions regarding what to include in the problem list and therefore the best common approach to the problem list is unknown. This knowledge would help the medical community move forward in developing such mechanisms.

The purpose of this study is to develop a better understanding of how practitioners think about and use the problem list. A secondary purpose is to study the extent to which practitioners differ in their decision making and if these decisions vary based on practitioner characteristics such as clinical work experience, specialty, and age. Such research will assist in the pursuit of developing policies and tools that can create a common approach to the problem list.

## Methods

To identify practitioner opinions towards the problem list and the logic behind their decisions an observational cross sectional study was conducted at Brigham and Women’s Hospital (BWH) and Massachusetts General Hospital (MGH). Of note, the EHRs at these facilities allow both coded and free text diagnosis to be entered into the problem list, providing the practitioners with great freedom in how they approach the problem list. This study was granted IRB exemption from the Partners HealthCare Human Subjects Committee.

### Study design

**Figure 1:**
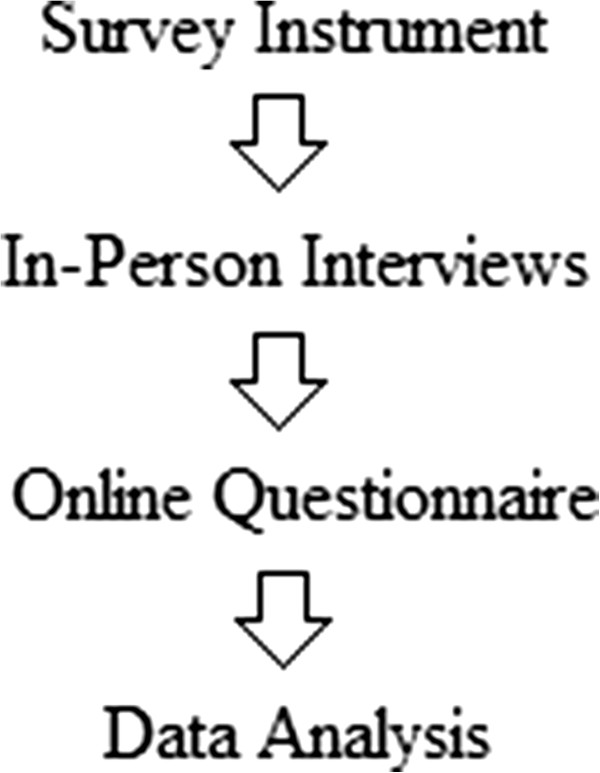
Mixed-methods study design order.

A survey instrument was created to identify practitioner attitudes towards the problem lists in areas that were predicted to be variable. Then the survey instrument was administered through a two-pronged approach. First, in person interviews were conducted with practitioners to understand the logic behind their hypothesized actions towards the problem list. Second, an online questionnaire was sent to practitioners to gain numerous viewpoints. For the data analysis stage, both data sets were used in conjunction to create a summarized analysis of practitioner opinions towards the problem list. These steps are outlined in Figure 1 below.

### Survey instrument

Based on the premise that well-meaning practitioners will differ in their views about actions towards the problem list, the survey instrument focused on areas of action towards the problem list that were thought to be highly variable across practitioners. These non-standardized areas were defined and categorized based on the prior research experiences of the study team. These experiences included a project to improve coded problems at a health services organization from a major research university [10,11], an ethnographic study of healthcare providers’ use of the problem list [6], and experience with developing an EHR tool which alerts practitioners to potential problem list gaps [17]. Based on the knowledge learned from these undertakings, the areas of focus for the survey instrument are defined and explained in Table 1.

**Table 1:** Details on problem list vignette questions

**Question**	**Category**	**Potential Controversy**	**Example**
**What problems should be included (Broad)?**			
	1 Family History	Should family history only be listed in the family history section of the EHR or if important enough be included in the problem list.	Family history of breast cancer; Family history of diabetes
	2 Social History	Should social history only be listed in the social history section of the EHR or if important enough be included in the problem list.	Construction worker; Non-smoker; Suspected alcohol abuse
	3 Surgeries	Should surgeries only be listed in the past surgical history section of the EHR or if important enough be included in the problem list.	Appendectomy; Knee replacement surgery
	4 Hospitalizations	Should hospitalizations only be listed in the prior hospitalization section of the EHR or if important enough be included in the problem list.	Hospitalized - May 2006 - MI
**What problems should be included (Detailed)?**			
	5 Latent chronic diseases	Should chronic diseases which are currently not receiving medical treatment be included in the problem list?	Asthma, no symptoms, no medications
	6 Non-medical conditions	Should problems that are not a disease, family history, social history, surgery, or hospitalization be included in the problem list?	Medical anxiety; Medication non-compliance
	7 Undiagnosed long term symptoms	Should symptoms that cannot be linked to a specific diagnosis of a disease be listed in the problem list?	Chest pain - work up completed, no diagnosable cause
	8 Multiple occurrences of transitive illness	Should transitive illnesses that occur multiple times be listed in the problem list?	Multiple urinary tract infections
	9 Sequelae problems	Should a disease caused by an original disease be listed in the problem list?	Coronary heart disease caused by diabetes
**Terminology**			
	10 Use of acronyms	Should practitioners use acronyms in the problem list or write out the full title of the disease?	DM or Diabetes Mellitus; CHD or Coronary Heart Disease
	10 Level of detail of problems	What level of specificity should be used to describe a problem?	Diabetes; Diabetes Mellitus; Diabetes Mellitus Type II
	11 Listing a sequelae	Should a problem caused by an original disease be listed with the original problem on the problem list?	Diabetes Mellitus Type II with renal manifestations
**When to add or delete problems?**			
	12 Timing (add)	On a problem where it is unclear if it is transitive or chronic, how much time or number of appointments should the practitioner wait until listing it on the problem list?	Back pain
	13 Timing (delete)	When a chronic disease is cured or no longer receives medical treatment, should it be deleted from the active status problem list and if so, when?	Diabetes Mellitus Type II; Breast Cancer; Migraines
**Sensitive Problems**			
	14 Whether to include sensitive problems?	Should sensitive problems be included in the problem list?	Depression, HIV/AIDS
	15 To include sensitive problems when other practitioners have access to the same record	Should all the patient’s practitioners know of all their diagnoses through the problem list in the EHR?	Anorexia Nervosa; HIV positive
	16 To include sensitive problems when a patient disagrees with a diagnosis	Should a diagnosis that a patient does not believe they have be listed in the problem list?	Depression; Anxiety Disorder
	17 To include sensitive problems when a patient has access to the problem list through an online patient portal	If a patient has access to their problem list through an online patient portal, should a diagnosis that could potentially hurt the patient’s feelings be listed on the problem list?	Obesity; Depression
**Who can change the problem list across the following roles:**			
	18 Specialist	Should specialists be responsible for adding or deleting problems that they diagnose or treat?	Asthma; Breast Cancer
	18 PCP	Should the PCP be solely responsible for adding and deleting all problems, regardless of who originally diagnosed the problem?	All potential problems
	19 Nurse Practitioner	Should a nurse practitioner be allowed to add and/or delete problems when they care for a patient?	All potential problems
	20 Other RN	Should an RN other than a nurse practitioner be allowed to add and/or delete problems when they care for a patient?	All potential problems

After determining the specific areas to study, the survey instrument was constructed to gather practitioner opinions. This survey instrument has two sections: the first part asked background questions such as clinical discipline, age, medical experience, and importance of the problem list to the respondent measured via a Likert scale. The second part of the questionnaire consisted of vignettes. Each vignette contained a hypothesized clinical scenario that covered one of the predefined areas under question along with multiple choice responses of potential actions. For instance, the question of whether family history should be included in the problem list was represented by the following vignette:

"Donna goes to see her PCP and mentions that she is terrified of getting breast cancer because both her maternal grandmother and mother had breast cancer. Now her sister was recently diagnosed. Should Donna’s family history of breast cancer be mentioned on her problem list? (Yes/No)"

*The full questionnaire is available in Additional file 1: Appendix A.

Vignettes were used in the survey instrument instead of asking direct questions (for example, “Do you include family history in your problem lists?”) because it was thought that practitioners would be challenged to think more deeply about their answers if given a real life scenario.

### Validation

To validate that the questionnaire was medically accurate and appropriate, the survey instrument was reviewed by a physician and pre-tested with a focus group. Focus group participants included physicians who were currently students at the Harvard School of Public Health. The survey was conducted through the Turning Technologies Audience Response System (Turning Technologies, Youngstown, OH) where each student anonymously responded to a vignette and the results were then displayed on the screen. The focus group leader then prompted a discussion asking individuals to describe their thought process behind their decision. Overall, the focus group respondents understood the vignettes, were engaged in the exercise, and had definite opinions about their answers.

After the validation process, the survey instrument was further refined to ensure questions were clear and reordered to place compelling questions at the beginning to encourage completion of the instrument.

### Sample

The survey instrument was then implemented via the prior described two-pronged approach: in-person interviews and online questionnaire.

For the in-person interviews, practitioners at both Brigham and Women’s Hospital (BWH) and Massachusetts General Hospital (MGH) were contacted to participate. A representative sampling method was not feasible and contacted practitioners were selected based on prior participation in a problem list study at the hospitals.

In-person interviews were conducted between December 2010 and February 2011. During the interviews, the interviewer followed the survey instrument. When answering the vignettes, the interviewer followed up with more specific questions to learn how the respondent justified their answer. All interviews were recorded for data tracking purposes. No incentives were provided to respondents. After the interview was conducted, practitioners were given a $5 Starbucks gift card as a thank you token. The practitioners were unaware of the gift card prior to the interview.

For the online questionnaire, the survey instrument was formatted and data collected using REDCap electronic data capture tools [18]. REDCap (Research Electronic Data Capture) is a secure, web-based application designed to support data capture for research studies. To address issues that arose during the in-person interviews, the survey instrument was slightly modified for use in the online questionnaire. Overall, it is not believed that the modifications affected the intent of the questions between the in-person interviews and online questionnaire.

The online questionnaire was hosted from March through June 2011. Physicians (including primary care providers and specialists), physician assistants (PAs), and nurse practitioners (NPs) were included in the sample. The online questionnaires were sent to several departments at BWH and MGH consisting mostly of primary care providers. The respondent pool was determined based on access to department electronic mailing lists which required approval by department directors. All surveyed departments received approval for participation in the study and only one invitation to a division of specialists received no response and thus did not participate in the study. No incentives were provided to respondents.

### Data analysis

Data collected through the in-person interviews were partially transcribed, partially summarized where appropriate. The same person who conducted the in-person interviews completed the transcription. In creating Additional file 2: Appendix B, the transcriptionist (CH) looked for quotes that represented the different justifications behind decisions towards the problem list in each vignette.

The data from the online interviews were aggregated and analyzed using STATA. The data were then tabulated to observe the proportion of responses to each answer. These proportions were combined with the quotes taken from the in-person interviews in order to create a general analysis of each area of non-standardized practice discussed in the results section [Additional file 2: Appendix B].

Using the responses to the online questionnaire, two summary measures were created using the following methodology:

● *Completeness measure:* Thirteen of the vignettes specify a situation where a problem is either added or not added to the problem list (Questions #8 - 16, 18, 19, 21, and 24 in Additional File 1: Appendix A). The completeness score displays how many problems the respondent wanted to add to the list across the vignettes. Respondents received one point for every time they selected “yes” to add a potential problem to the problem list. Respondents received zero points for every time they selected “no” to keep a potential problem off the problem list.

● *Plurality measure:* This measure was designed to see how opinions of individuals differed from the group. Respondents who answered with the relative majority received one point. Respondents who answered with any other response received zero points. For vignette question with more than two responses, respondents received one point if they answered with the response that received the highest percentage of respondents (the plurality) and zero points if they answered with any of response. An aggregate measure was then calculated for each respondent. All vignette questions were analyzed in this measure unless the question responses were evenly split and no plurality existed.

These two summary measures were tabulated and summarized within STATA. We used Student’s *t* test to compare scores based on provider characteristics such as:

● Discipline (MD, PA/NP, Other)

● Role (PCP, Specialist, Other)

● Training status (Resident or Non-Resident)

● Age

● Years of experience practicing medicine

● Importance of the problem list to everyday practice of medicine conducted via a Likert scale of one “not important” to five “very important.”

In addition to Student’s *t* test, we also used linear regression models to explore the combined effects of these characteristics in a multivariate setting. P values were used to determine statistical significance measured at the 95% confidence level.

## Results

### Response rate

Invitations to complete the online questionnaire were sent out to 346 practitioners. The online questionnaire received ninety-seven full responses and fourteen partial responses (response rate: 32%). For the in-person interviews, we contacted fourteen and completed nine interviews (response rate: 64%), ranging in length from eighteen to forty-three minutes (Table 2). One interview had to be excluded from further analysis as the practitioner did not feel confident in his ability to appropriately answer the vignettes. The other eight in-person interviews were completed with practitioners across a variety of disciplines and experience levels (Tables 3 and 4).

**Table 2:** Summary of respondents by data collection type

	**n**	**Sample size**	**Response rate**
In-Person Interviews	9*	14	64.2%
Online Questionnaire	111	346	32.1%

*One survey response was excluded from further analysis.

**Table 3:** **Online questionnaire respondents, and linear regression and *t*-test analysis of the completeness and plurality measures**

	**Online Questionnaire Respondents**		**Completeness Measure**		**Plurality Measure**	
			**Assessment of 13 Yes/No vignettes for inclusion tolerance**		**Assessment of 20 vignettes for trends in choosing with the plurality**	
	**N**	**% Resp**	**Unadjusted Value**	Adjusted P*	**Unadjusted Value**	Adjusted P*
**Role**						
MD	105	93.8	9.7	ref	15.1	ref
PA/NP	6	5.4	10.8	0.5	14.6	0.3
Other	1	0.9	NA	NA	NA	NA
**Discipline**						
PCP	85	76.6	9.7	ref	15.0	ref
Specialist	9	8.1	9.9	0.9	15.3	0.7
Other	17	15.3	10.2	0.2	15.0	0.6
**Resident**						
No	48	44.0	9.9	ref	15.0	ref
Yes	61	56.0	**9.4**	**0.0**	15.0	0.7
**Age**						
21-30	38	34.9	9.5	ref	15.1	ref
31-40	27	24.8	9.5	1.0	14.6	0.7
41-50	23	21.1	9.9	0.5	15.1	0.1
51-60	13	11.9	10.3	0.7	15.4	0.1
61-70	7	6.4	10.0	0.9	15.3	0.1
greater than 70	1	0.9	**12.0**	**0.0**	15.0	0.1
**Experience**						
less than 1 year	20	18.4	9.9	ref	15.6	ref
1-5	34	31.2	9.3	0.1	14.9	0.3
6-10	10	9.2	9.4	0.1	14.8	0.3
11-20	24	22.0	9.9	0.5	**15.0**	**0.0**
21-30	11	10.1	10.5	0.9	14.8	0.1
31-40	7	6.4	10.0	0.4	**15.3**	**0.0**
greater than 40 years	3	2.8	11.0	0.6	15.7	0.3
**Importance**						
1 - not important	2	1.8	10.0	ref	14.5	ref
2	3	2.7	10.5	0.3	**17.5**	**0.0**
3	10	9.0	9.5	0.9	14.8	0.5
4	37	33.3	9.7	0.2	15.0	0.1
5 - very important	59	53.2	9.8	0.4	15.0	0.2

**Table 4:** Characteristics of in-person interview respondents

	**% Resp**	**N**
**Role**		
MD	87.5%	7
RN/NP	12.5%	1
**Discipline**		
PCP	25.0%	2
Specialist	75.0%	6
**Resident?**		
Yes	12.5%	1
No	87.5%	7
**Experience**		
1-5	12.5%	1
6-10	12.5%	1
11-20	25.0%	2
21-30	37.5%	3
31-40	12.5%	1

### Characteristics of respondents

Table 3 describes the characteristics of our online questionnaire respondent base. Most of the respondents were physicians (94%) and the second most frequent respondents were physician assistants/nurse practitioners (5%). The questionnaire was programmed to terminate responses from those who selected other (1%) to ensure that all respondents had a clinical background.

Most of the respondents were PCPs (77%), and nearly half of the respondents were residents. The distribution of age closely followed the distribution of experience among respondents. Demographic data sets were not available for non-respondents in departments that agreed to participate or the one department that was invited to participate in the questionnaire, but did not respond to the invitation.

Table 4 includes the demographic data of respondents to the in-person interviews. 88% were physicians and among this group, 75% were specialists. Experience varied widely and only one respondent was a resident.

### Character of responses

Additional file 2: Appendix B includes select quotes from the in-person interviews. The quotes show some of the logic and opinions behind potential answers to the vignettes. The tabulated data from the online questionnaire are included for convenient reference. Overall, the in-person interviews brought out the complexities behind contentious issues in creating the problem list.

Table 5 includes the tabulated answers to each individual vignette from the online questionnaire. Of the thirteen vignettes with a yes or no response, twelve had a meaningful majority answer (statistically significant based on a binomial distribution). The one question that was evenly divided covered whether hospitalizations should be included in the problem list (question4 respectively in Table 5). The other seven questions that contained non-yes/no responses (questions #10, 11, 12, 13, 18, 19, & 20 in Additional File 1: Appendix A) had a range of answers depending on the question.

**Table 5:** Tabulated answers to vignettes

**What problems should be included (broad)?**			
**Question 1: Family History**	**Answers**	**% Res.**	**N**
Donna goes to see her PCP and mentions that she is terrified of getting breast cancer because both her maternal grandmother and mother had breast cancer. Now her sister was recently diagnosed. Should Donna’s family history of breast cancer be mentioned on her problem list?	Yes	76.2	77
	No	23.8	24
**Question 2: Social History**	**Answers**	**% Res.**	**N**
John comes in to a medical center's urgent care ward with a small facial laceration from playing hockey. John mentions he's a male model to the physician and explains that he wants treatment that will minimize scarring. Should the doctor add John's occupation as a model to the problem list?	Yes	7.9	8
	No	92.1	93
**Question 3: Surgeries**	**Answers**	**% Res.**	**N**
Ritchie has an appendectomy performed at the local hospital. His PCP gets the medical record from the hospital. Should Ritchie's PCP add 'appendectomy' to Ritchie's problem list?	Yes	73.5	72
	No	26.5	26
**Question 4: Hospitalizations**	**Answers**	**% Res.**	**N**
Paul is hospitalized due to a heart attack caused by his coronary artery disease. At Paul's PCPs office 'coronary artery disease' is already listed on his problem list. Now Paul's PCP receives Paul's medical information from the hospital. Should Paul's PCP add another item specifically mentioning Paul's recent hospitalization to the problem list?	Yes	50.0	49
	No	50.0	49
**What problems should be included (detailed)?**			
**Question 5: Latent non-transitive diseases**	**Answers**	**% Res.**	**N**
Tenesha recently moved to Boston and goes to see her new PCP for an annual physical. Tenesha says that she was diagnosed by a pulmonologist with exercise-induced asthma several years ago. Currently, she takes no medications to treat her asthma, is experiencing no symptoms, and the asthma does not affect her daily life. Should 'exercise-induced asthma' be added to her problem list?	Yes	82.2	83
	No	17.8	18
**Question 6: Non-medical conditions**	**Answers**	**% Res.**	**N**
Maria is a 52 year old woman and is afraid of doctors. She summons up the courage to go see a doctor for the first time in years because of a persistent cough. Should the doctor add a note about Maria's fear of doctors to her problem list?	Yes	35.6	36
	No	64.4	65
**Question 7: Undiagnosed long term symptoms**	**Answers**	**% Res.**	**N**
Jorge appears to have ongoing chest pain, but after a full work up the practitioner cannot diagnose the cause. Should the practitioner add an item about chest pain to the Jorge's problem list?	Yes	96.0	97
	No	4.0	4
**Question 8: Multiple occurrences of transitive illness**	**Answers**	**% Res.**	**N**
Helen is having her third urinary tract infection (UTI) within one year. Should the practitioner add a statement about the Helen's predisposition for urinary tract infections to her problem list?	Yes	92.9	91
	No	7.1	7
**Question 9: Sequelae**	**Answers**	**% Res.**	**N**
Sally develops coronary artery disease as a result of her Type II diabetes. Should the resultant coronary artery disease be listed on the problem list?	Yes	100.0	97
	No	0.0	0
**Terminology**			
**Question 10: Use of acronyms/Level of detail of problems**			
Sally is diagnosed with Type II diabetes. What term should the practitioner use on Sally’s problem list?			
**Answers**	**% Res.**	**N**	
DM	1.0	1	
DM II	16.5	16	
Diabetes Type II	17.5	17	
Diabetes	3.1	3	
Diabetes Mellitus	1.0	1	
Diabetes Mellitus Type II	58.8	57	
Other	2.1	2	
**Question 11: Listing of sequelae**			
If a practitioner wants to list Sally's coronary artery disease, how should coronary artery disease be listed on her problem list?			
**Answers**	**% Res.**	**N**	
Diabetes Type II with coronary artery disease	0.0	0	
As a separate problem from Diabetes Type II	100.0	97	
**When to add or delete problems?**			
**Question 12: Timing (add)**	**Answers**	**% Res.**	**N**
Dr. Baker likes to include long term undiagnosed symptoms on his patients' problem lists. Catherine comes in for her first appointment with Dr. Baker complaining of lower back pain. If Catherine keeps coming to see Dr. Baker once a month complaining of lower back pain, at what appointment/month should Dr. Baker add an item about back pain to Catherine's problem list?	1st appointment/month	27.6	27
	2nd	12.2	12
	3rd	25.5	25
	4th	3.1	3
	5th	0.0	0
	6th thru 11th	4.1	4
	> 12th	0.0	0
	It depends	26.5	26
	Never	1.0	1
**Question 13: Timing (delete)**	**Answers**	**% Res.**	**N**
The practitioner does mention Helen's predisposition for UTI's on her problem list. Three months later, Helen is in for her annual physical and mentions that she has not had any UTIs for the past three months. Helen continues not to experience anymore UTIs. At what point should the item about Helen's predisposition for UTIs be removed from the problem list?	1-3 months	6.1	6
	4-6 months	4.1	4
	6-9 months	5.1	5
	10-11 months	4.1	4
	1-2 years	38.8	38
	3-4 years	6.1	6
	>5 years	2.0	2
	Never	8.2	8
	It Depends	25.5	25
**Sensitive Problems**			
**Question 14: Whether to include sensitive problems?**	**Answers**	**% Res.**	**N**
Paul goes to see a psychiatrist and is diagnosed with depression. Should the psychiatrist add 'depression' to Paul's problem list?	Yes	99.0	97
	No	1.0	1
**Question 15: To include sensitive problems when other practitioners have access to the same record**	**Answers**	**% Res.**	**N**
Janice goes to see a psychiatrist and is diagnosed with anorexia nervosa. She also goes to see a PCP, allergist, gynecologist, and neurologist at the same medical facility. While her psychiatrist's notes are restricted to the mental health department, all of Janice's other doctors are viewing a common problem list through an electronic health record (EHR) system. Under this scenario, should the psychiatrist add 'anorexia nervosa' to Janice's problem list?	Yes	99.0	97
	No	1.0	1
**Question 16: To include sensitive problems when a patient disagrees with a diagnosis**	**Answers**	**% Res.**	**N**
Dr. Thomas works at a mental health facility that encourages psychiatrists to add mental health problems to the patients' problem lists. During one of Dr. Thomas's patient visits, the patient strongly disagrees with the diagnosis of depression. Should Dr. Thomas still list 'depression' on the patient's problem list?	Yes	75.5	74
	No	24.5	24
**Question 17: To include sensitive problems when a patient has access to the problem list through an online patient portal**	**Answers**	**% Res.**	**N**
Dr. Brown works at a health center that offers their patients the ability to view their entire electronic health record online through a patient portal. Dr. Brown is with a patient whom he diagnoses with obesity. Dr. Brown knows this patient regularly checks the patient portal to review her medical record. Should Dr. Brown list 'obesity' on this patient's problem list?	Yes	93.9	93
	No	6.1	6
**Who can change the problem list across the following roles:**			
**Question 18: Specialist/PCP**			
Toby appears to have an asthma attack during a soccer game. His PCP refers him to a local pulmonologist. The pulmonologist diagnoses him with asthma and has access to the same electronic health record as the PCP. How should the pulmonologist address the problem list?			
**Answers**	**% Res.**	**N**	
The pulmonologist should add 'asthma' to Toby's problem list.	77.8	77	
The pulmonologist should advise the PCP to add 'asthma' to the problem list in his follow up.	9.1	9	
The pulmonologist should perform his regular feedback and assume the PCP will add 'asthma' to Toby's problem list if the PCP feels it is necessary.	12.1	12	
Other	1.0	1	
**Question 19: Nurse practitioner**			
John recently moved to Boston and is going for his annual physical exam with a new health center. A nurse practitioner is giving John his physical exam and John tells the nurse practitioner that he was diagnosed with asthma by a pulmonologist. Should the nurse practitioner be able to add problems like John's asthma to the problem list or should only physicians be able to add problems?			
**Answers**	**% Res.**	**N**	
The nurse practitioner should be able to add problems to the problem list.	93.8	91	
Only physicians should be able to add problems to the problem list.	6.2	6	
Other	0.0	0	
**Question 20: Other RN**			
Carlos breaks his leg and goes to the hospital. The nurse is performing her medication rounds when Carlos mentions to her that he forgot to tell the triage nurse that he has hemophilia. What should the nurse do in regards to the problem list?			
**Answers**	**% Res.**	**N**	
The nurse should access Carlos's medical record and add 'hemophilia' to the problem list.	22.7	22	
The nurse should tell the doctor that Carols has hemophilia and recommend that the doctor add 'hemophilia' to the problem list.	60.8	59	
The nurse should tell the doctor that Carols has hemophilia and assume that Carlos's doctor will add 'hemophilia' to Carlos's problem list without specific recommendation.	14.4	14	
Other	2.1	2	

### General trends and findings

The following represents a brief summary of the findings and trends in each category as described in the methodology.

#### What to include in a problem list (broad categories): questions #1-4

This category covers whether content such as family history, social history, surgeries, and hospitalizations should be included in the problem list [Table 5 and Additional file 2: Appendix B]. According to the survey responses, a strong majority of practitioners answered for the family history (Question #1, Yes: 76%) and surgeries (Question #3, Yes: 73%) to be included on the problem list. Hospitalizations were contentious at an exact 50/50 split (Question #4). Further, most practitioners (92%) rejected adding an occupation to the problem list (Question #2).

The in-person interviews revealed that practitioners want this information to be easily accessible, but it can be in their own separate list in the medical record or categorized within the problem list. The biggest concern was adding these factors in both locations, creating redundancies across the medical record and extra work for the provider.

A key question that intrigued the interviewed practitioners was Donna’s strong family history of breast cancer (Question #1). Some practitioners agreed the family history was important enough to be on the problem list, regardless if it was also included in the family history section of the EHR. For social history, (Question #2) practitioners were concerned that adding an occupation to the problem list would clutter the list as the factor would be unlikely to affect future care or in the specific vignette where the occupation was “male model”, perpetuate a negative stigma. One practitioner discussed that she would want to see an occupation listed if it correlated strongly with exposure to a known health hazard.

#### What to include on a problem list (detailed inclusions): questions #5-9

The category covers more finely detailed specifications for inclusion of problems. From the online questionnaire, practitioners showed that they are not limited to the strictest definition of a problem list; namely, chronic diagnoses. Based on the online survey data, practitioners want to include latent chronic diseases (Question #5, Yes: 82%), undiagnosed long term symptoms (Question #7, Yes: 96%), multiple occurrences of transitive illnesses (Question #8, Yes: 93%), and sequelae of problems (Question #9, Yes: 100%). Non-medical conditions (Question #6) or the inclusion of the women’s fear of doctors was more controversial (Yes: 36%).

In-person interviews showed that inclusion of these detail specific issues are not as straightforward as the online questionnaire displayed and the action often depends on the patient. For example, in response to question #5 about the woman diagnosed with asthma that was taking no medications and experiencing no symptoms, one practitioner responded that for a young person with few health problems it would not be an issue to add asthma to the problem list. Yet, if the patient was older with multiple health problems and consequently a lengthier problem list, he would be less likely to add the problem. At some point practitioners start editing for length to avoid missing the most crucial facts about the patient the next time they review the problem list.

On question #6 or the vignette about the women’s fear of doctors, some interviewed practitioners expressed that their care would remain unaffected by knowing this information and felt its inclusion in the problem list perpetuated a negative stigma. Other practitioners wanted to know such information upfront because it would affect their interpretation of the medical record and they did not want this information buried within the medical note. This example shows how well-meaning practitioners can differ in their action towards the problem list depending on how they wish to practice medicine.

#### Terminology: questions #10-11

This category covers how problems should be listed in the problem list. It is important to note that the respondents to these questions are allowed to add structured or free text problems to their patient’s problem lists in the EHR and therefore have an active choice in their day to day practice of medicine to use personal preferences in listing terminology.

From analysis of the online questionnaire, practitioners indicated that they wished to include more detail in the language used on the problem list than less (Question #10). Only 5% of respondents selected a phrase for diabetes that did not specify type II. Usage of acronyms showed more variability with 16% selecting the equivalent acronym (DM II) and 59% selecting the unabbreviated phrase (Diabetes Mellitus Type II). Finally, respondents unanimously agreed (100%) that sequelae of an initial problem should be listed as a separate problem (Question #11).

Based on the in-person interviews, it appeared that this issue was placed between the ideal world scenario (complete phrases) and reality (practitioners still commonly use acronyms). On sequelae of problems, one practitioner added a notable bit of clarity that once a problem generates its own medical care it should be listed as a separate problem.

#### When to add or delete problems: questions #12-13

This category covers issues of timing, specifically when practitioners should add problems. The category also includes if practitioners should remove resolved or inactive problems from active status on the problem list and if so, when.

In the online questionnaire, practitioner responses of when to add a transitive problem (Question #12) were clustered around the first three medical visits focused on the health issue (first: 28%, second: 12%, and third: 26%). Opinions on if and when to remove a problem appeared more varied (Questions #13). The highest proportion of responses (39%) was for removing a problem after 1–2 years of non-occurrence. The answer “it depends” received the second highest proportion of responses (26%). Responses were relatively evenly distributed across all other answers which included time periods such as “1-3 months,” “4-6 months” and so on up to “Greater than 5 years.” Only 8% of respondents selected the option “never”.

In general, practitioner responses during the in-person interviews correlated strongly with the online questionnaire (somewhere within the first several visits or months). One practitioner discussed that he was hesitant to add a transitive problem at the first encounter as he found many patients complained of a symptom in the first visit such as back pain and proceeded never to mention it again.

When asked about removing a problem from the list, practitioners explained that it was a complex yet important issue. Answers centered around 1–2 years like the online questionnaire responses, but often came with qualifications. One practitioner mentioned that the lack of specific guidelines and mechanisms for removing cured or latent problems from the list was a key cause of lengthy, cluttered, and unreliable problem lists.

#### Sensitive problems: questions #14-17

This category covers the inclusion of sensitive problems on the problem list such as mental health conditions and HIV status, particularly in scenarios where such information is more easily distributed through information technology.

On inclusion of more sensitive problems (Question #14), practitioners nearly unanimously responded to the online questionnaire that they should be included (Yes: 99%). Further, practitioners did not feel the inclusion of the problem should be affected in situations where the problem list is shared across multiple practitioners (Question #15, Yes: 99%), a patient disagrees with the diagnosis (Question #16, Yes: 76%), or the patient views their record online (Question #17, Yes: 94%).

Some practitioners expressed during in-person interviews that leaving sensitive problems off the problem list would perpetuate negative stigmas and omit crucial information that other practitioners need to know in order to give the best care.

In the vignette where patients were viewing their records online, the interviewed practitioners discussed changing the language to avoid the use of more negative terms. For instance, one practitioner expressed that obesity could be written as a BMI measure. Also, there was a clear concern over maintaining legal privacy of patient health information. One practitioner stated that in an ideal world she would want to have sensitive problems included on the problem list, but she was unaware if the inclusion was in compliance with Health Insurance Portability and Accountability Act (HIPAA) privacy rules. In general, practitioners were aware that special HIPAA standards for treatment of sensitive information existed. Yet, the practitioners were unsure how they would apply to situations where such information was shared through a problem list in the EHR.

#### Who can change the problem list?: questions #18-20

This category covers the issue of who can add problems to the problem list and also who is ultimately responsible for problem list maintenance such as reviewing, updating, and deleting problems. The issue traditionally is a debate between PCPs and specialists who are both diagnosing new problems and have access to the same problem list.

Among respondents to the online questionnaire, 78% answered that the specialist should put a problem they diagnose on the problem list (Question #18). There appeared to be little issue with nurse practitioners adding problems to the problem list (Question #19, 94%). However, only a minority of respondents endorsed an inpatient nurse adding a problem to the problem list (Question #20), with the majority (61%) preferring that the nurse alert the physician to the problem with a recommendation to add it to the problem list.

Interviewed practitioners discussed that a specialist adding to the list is likely an ideal world scenario not currently met in medical practice at the study sites. Some specialists expressed that adding a problem to the problem list would be an incursion on an area of the medical record owned by the PCP, or that adding to the problem list is extra work that is not strongly relevant to the care they provide. The PCPs interviewed said that they would like to see specialists add to the problem list. For one specialist, he saw this issue as the key to why problem lists are often incomplete and therefore unreliable to use in medical practice.

On the role of nurse practitioners, interviewed practitioners expressed that it was essential for nurse practitioners to make changes to the problem list, particularly if we have the goal to maintain complete and accurate lists. On the inpatient nurse, a medical resident reported that nurses and doctors do not currently add new diagnoses to the problem list during inpatient care, thus is not highly relevant given current behavior. This issue requires more research and a larger sample size to explore the entirety of inpatient care attitudes towards the problem list and the viewpoints on inpatient nurses adding to the list.

### Analysis of the completeness and plurality measures

Table 6 includes the tabulated results of the completeness and plurality measures. Summary data for the completeness measure ranged from six to twelve with a mean of 9.7 and standard deviation of 1.4. Overall, nearly 50% of respondents received a score of 9 or 10. With a maximum possible completeness score of thirteen, most practitioners voted together on placing more on the problem list than less.

**Table 6:** Tabulation of completeness and plurality measures

	**% Resp**	**N**
**Completeness Measure**		
6	1.1%	1
7	2.1%	2
8	18.1%	17
9	23.4%	22
10	23.4%	22
11	19.2%	18
12	12.8%	12
**Plurality Measure**		
11	1.1%	1
12	4.3%	4
13	16.0%	15
14	17.0%	16
15	19.2%	18
16	22.3%	21
17	13.8%	13
18	6.4%	6

The plurality measure ranged from eleven to eighteen with a mean of 15 and standard deviation of 1.7. As question four on inclusion of hospitalization history received an exact 50/50 response there was no plurality and data from this question was not included in the analysis of the plurality measure. With a maximum possible score of nineteen, the mean of 15.0 indicates that there is a strong amount of homogeneity in practitioner responses across the questions, but elements of heterogeneity still exist.

Finally, Table 3 includes the results from the univariate and multivariate analysis of the completeness and plurality measures against the demographic data. Only a few significant trends were found among the measures as indicated below.

Completeness Measure:

● Non-residents answered with more “yes” responses to adding problems to the problem list than residents (data from the 13 vignette questions with strictly “yes” or “no” responses).

● Practitioners over 70 years of age were more likely to answer “yes” to adding problems to the problem list than practitioners of 21–30 years of age.

Plurality Measure:

● Practitioners with 11–20 years of medical experience were less likely to answer with the plurality than practitioners with less than 1 year of experience.

● Practitioners with 31–40 years of medical experience were less likely to answer with the plurality than practitioners with less than 1 year of experience.

● On the measure of problem list importance, those who selected answer two “less important” were more likely to vote with the plurality than those who selected answer one “not important”.

## Discussion

### Support for policy and tools

This study confirmed the hypothesis that practitioners differ in their opinions over what should and should not go on the problem list, although many areas of agreement were identified. This difference in opinion is likely a key reason for the variation in the content and structure of current problem lists within and across healthcare organizations. Without consistency across problem lists, patients cannot receive the full benefits problem lists bring to patient care, namely better practitioner compliance with best practices and the complete utilization of clinical decision support and population management tools. The medical community needs to work towards standardization through the development of policies about how the problem list should be used as well as tools built into the EHR that can help practitioners comply with those policies.

Unlike prior research, one valuable component to the study is that the online questionnaire provided quantitative evidence about the size of the disagreement over actions towards the problem list. For instance, all but one vignette question held a statistically significant plurality suggesting that a large portion of practitioners are approaching the problem list in a similar manner. This result implies that it is possible for a majority of practitioners to agree on a common approach to the problem list.

The data also brings attention to possible differences among PCP’s and specialists concerning problem list “territory.” While specialists believe adding to the problem list would be an incursion on an area of the medical record owned by the PCP, the data indicates that PCP’s believe specialists should feel comfortable adding to the problem list.

The findings show that the problem list needs more functionality to help practitioners contribute to the document and also make the list more useful to their work. For instance, one specialist spoke about how when he stages a patient for breast cancer (entering data about the exact size and shape of the tumor) he is frustrated that the EHR cannot follow the logical consequence of automatically generating “breast cancer” on the problem list. Here is an instance where tools could help make the problem list a more integrated part of the practitioner’s medical practice and also make it easier to comply with any future policies.

Finally, the summary measures showed a weak correlation between opinions towards the problem list and any common grouping characteristics such as age, medical experience, or opinion on the importance of the problem list. For the completeness measure, the only significant factor with the support of a decent sample size was that residents wanted for less to be included on the problem list than non-residents. Speculation on these differences could be changes to recent training or less experience in the medical field. Although it is important to note that no significant differences were found for the completeness measure amongst the experience and age variables.

The plurality measure contained several more significant measures, specifically within the experience and importance categories. These data indicate that practitioners may approach the problem list differently than their peers based on these characteristics. Yet, with no true dose response and the smaller sample size, this premise is certainly not conclusive and requires further study. In developing a common approach to problem list, these data are not strong enough evidence to suggest value in segmenting opinions by common demographic factors.

### Recommendations

Based on the in-person interviews, several issues stood out as greater challenges for practitioners than others. The following recommendations are areas that would benefit the most through the development of policies and EHR tools.

A major cause of unreliable problem lists is the general disagreement in the medical community over “Who is responsible for the problem list?” As discussed in the results section, the debate centers on the roles and responsibilities of the specialist versus the PCP. Due to the disagreement in responsibility, problems diagnosed by specialists do not have a consistent pathway onto the problem list. This process gap could be a primary cause of incomplete problem lists. An official ownership policy would bring clarity to the PCP and specialists relationship towards the problem list. For example, when asked what would need to change for the specialist to start having a more active role in the problem list, an oncologist responded:

"“If the [administration] came out and said everybody owns the problem list…if you are taking care of a problem then you need to make sure that problem is on the problem list, and then I think I would go ahead and do it.”"

Another potential cause of clutter and absent problems is the lack of guidelines for when to review the problem list and remove a cured or latent problem. A clarified policy could help specify when and who should be conducting this process and also the role the patient might play in reviewing their own problem list for accuracy. Removing inactive problem would help keep problem lists up-to-date, short, and relevant. Of note, such a policy would likely need to be closely intertwined with an ownership policy.

An additional cause of absent problems (or potentially worse offences) is the potentially murky understanding over how privacy and security regulations apply to the electronic problem list. In response to question #15 about placing sensitive questions on a problem list that can be viewed by many of the patient’s practitioners, one specialist commented:

"“I don't know what the rules are under this, but I think the diagnosis is relevant to everybody else. So the question is I don't know what the legality of mental health records is and how visible they are is, but that is where I would defer to someone and say I don't know. If there's a way where it’s not illegal to disclose that, then absolutely.”"

Concerns towards maintaining compliance with HIPAA and other privacy policies may be keeping practitioners from adding problems. Currently, HIPAA does not restrict what can be placed in the medical health record and instead regulates use and disclosure [19]. Discussions and clarification on how privacy can be maintained in the new digital age where problem lists are more readily accessed and available may help practitioners be more confident in their actions towards the problem list. The need to address this issue will likely become even more important as adoption of health information exchanges and online patient portals increases as well as the sensitivity of information evolves such as questions over listing genetic predispositions on the problem lists based on genetic testing.

Finally, this research gave insight into how restrictive a problem list policy should be towards allowing the addition of a broad range of problem types. As shown by the completeness measure, practitioners were more likely to want to include an item on the problem list than not. Throughout the study, practitioners did not limit themselves to the strictest definition of the problem list, namely only including chronic diagnoses. The results also indicated that any common approach to the problem list will need to leave room for the practitioners’ personal judgment. For instance, practitioner reactions during in-person interviews differed greatly to the question about the woman who was highly afraid of doctors. Some practitioners found the information irrelevant to how they would treat her and others wanted it to be the first fact they knew because it could potentially change their analysis of her health history. Variation in how practitioners use the problem list does have policy implication. Based on these findings, practitioners are not looking for a highly restrictive policy that restricts personal judgment on what should be included on the problem list.

Of course, the idea of an unrestrictive policy is not to say that problem lists should include every possible problem without regards to length. An “all inclusive” policy will not create problem lists that are easily scanned and make known the most essential health facts about patients. Further, when and what to include often depends on the patient. As one practitioner responded to the vignette about if a case of asymptomatic asthma should be listed on the problem list:

"“Yeah, that is a grey area, actually. From someone who is… you know… completely well. This is her only issue then I can see why this might make it on to the problem list. Young person. If it’s… you know… you’re going to be adding on to a list of 10 or 15 problems on a chronically ill person where this is not likely to be a big issue for her, then I could see where you wouldn’t put it on the list. The length of a list actually becomes an issue, I think, just like fatigue…attention fatigue.”"

Of course, the idea that there needs to be some moderation in the content included in the problem list was known prior to the research, this study showed that practitioners are not looking to be restricted to certain types of information such as only diagnosed diseases. They want the option to include anything, which leaves the greater challenge of how policies and tools can help prioritize information to create the most effective problem lists.

### Study strengths and limitations

A key strength of the study was the usage of vignettes to help reveal practitioners’ attitudes towards the problem list. While not measured, practitioners appeared to easily comprehend, debate, and find answers to their preferred action and the resulting data did not appear hindered by the vignettes. One downside of the vignettes is that they were narrowly defined to specific clinical situations. Other limitations with the survey instrument included inconsistency of the survey instructions with the vignettes. Specifically, the initial instructions requested that respondents answer in the perspective of a PCP while some of the questions requested the specialist perspective.

The second core strength was the use of the two-pronged implementation method of the survey instrument as it brought out both breadth and depth to analyzing practitioners’ opinions towards the problem list. The main weakness was the sample. It was limited to practitioners at two affiliated, well-resourced, academic medical centers in Boston where the EHRs allow great freedom in what can be entered into the problem list (both coded and free text problems). Further weaknesses in the sample were that the respondents came from sources within the healthcare centers that were opportunistic rather than representative. This convenience sample resulted in disproportionate respondent demographics. For instance, the online questionnaire sample consisted mostly of PCPs due to the departments asked to participate, and the in-person interviews consisted mostly of specialists. The results would likely be affected by having a more representative sample of practitioners from across the United States.

In regards to the online questionnaire, it became known after the questionnaire was sent that a limited number of non-clinicians were included in the department mailings lists such as administrative assistants and they likely received emails containing links to the questionnaire. As the survey was designed to screen out non-clinicians based on the response to the first question, the responses were not impacted as long as the non-clinicians answered honestly, but the true response rate is likely slightly lower than reported.

The unavailability of demographic data for non-respondents of the online questionnaire further limited the study sample as it is unknown if the respondents held similar opinions to the non-respondents. Further, lack of significant respondents in the specialist category limited the ability to identify differential response rates based on clinician factors. Increasing the sample size would also further strengthen the findings of this study, and create a more representative collection of data. A larger sample size would also allow for further analysis of differing opinions by various demographic factors, such as clinician specialty, and inpatient versus outpatient practice settings, on clinician attitudes towards the problem list. The results of this study are also specific to the capabilities and design of Partner’s Longitudinal Medical Record system used at the study sites. Yet, a core strength of surveying this particular provider population is that the Partner’s Longitudinal Medical Record system allows for both structured and free text input. This unique environment means that providers have an active choice in what they list in their problem list on a daily basis and therefore, could readily give feedback on their preferences to this study. Providers who are only allowed structured problems may not have a strong idea of what they would prefer to include or not include on the problem list due to working in a more regulated EHR system.

## Conclusions

An accurate and reliable problem list could provide great benefits to patient care through ensuring practitioners are aware of the most important health factors about a patient and creating a more refined database from which to identify disease-specific populations. This study showed that practitioners do differ in their judgment towards the problem list overall, but in most situations there is a common approach among a majority. Further, practitioners showed that they do not want to be highly restricted on what information can go on the problem list and that there are areas where they are not meeting their ideal actions towards the problem list. The creation of a policy to help guide a common approach as well as tools to encourage upkeep would be helpful in creating accurate problem lists over time. With a number of more detailed insights into practitioner opinions towards the problem list, this study provided a stronger foundation from which the healthcare community can move forward to improve the problem list to enable better patient care.

## Competing interests

The authors declare that they have no competing interests.

## Authors’ contributions

CH created the questionnaire, conducted interviews, collected online responses, transcribed and analyzed the data, and drafted the manuscript. MB participated in designing the study and drafting of the manuscript. DS participated in the review and redrafting process. AW participated in the design and coordination of the study, recruitment of respondents, data analysis, and drafting of the manuscript. All authors read and approved the final manuscript.

## Pre-publication history

The pre-publication history for this paper can be accessed here:

http://www.biomedcentral.com/1472-6947/12/127/prepub

## Supplementary Material

Additional File 1 **Appendix A. Problem List Questionnaire.** Description of Data: Questionnaire of the problem list vignettes used for the in-person interviews and online survey Click here for file

Additional File 2 **Appendix B. In-Person Responses to the Vignettes.** Description of Data: Tabulated data from online questionnaire and relevant quotes from the in-person interviews. Click here for file
