# Supplementary material for: Healthcare provider attitudes towards the problem list in an electronic health record: a mixed-methods qualitative study
Source: BMC Med Inform Decis Mak. 2012 Nov 11;12:127. doi: 10.1186/1472-6947-12-127 (PMC3534408; doi:10.1186/1472-6947-12-127)
Supplement: Additional File 1 — Appendix A. Problem List Questionnaire. Description of Data: Questionnaire of the problem list vignettes used for the in-person interviews and online survey [file 1472-6947-12-127-S1.pdf]

Problem List Questionnaire

Dear Practitioner,

Thank you for taking this survey on the problem list. The following contains several demographic questions that will be followed by a series of clinical scenarios relating to decisions about the problem list.

The survey should take 5-10 minutes to complete. Thank you for participating in this study.

1) Please list your discipline in the health care field?
- ☐ MD
- ☐ PA
- ☐ RN/ NP
- ☐ Other

2) Are you a PCP or specialist?
- ☐ PCP
- ☐ Specialist
- ☐ Other

3) Are you a medical resident?
- ☐ Yes
- ☐ No

4) What is your age?
- ☐ less than 21
- ☐ 21-30
- ☐ 31-40
- ☐ 41-50
- ☐ 51-60
- ☐ 61-70
- ☐ greater than 70

5) How many years have you been practicing medicine?
- ☐ less than 1 year
- ☐ 1-5
- ☐ 6-10
- ☐ 11-20
- ☐ 21-30
- ☐ 31-40
- ☐ greater than 40 years

6) What is your definition of a problem list? ____________________________________

7) On a scale of 1 - 5 with 1 meaning not important and 5 meaning very important, how important is the problem list to your everyday practice of medicine?
- ☐ 1 - not important
- ☐ 2
- ☐ 3
- ☐ 4
- ☐ 5 - very important

Next is a series of short scenarios relating to decisions about the problem list.

If you are a PCP, please answer each question based on what you believe you would do as the clinician in each scenario. If you are a specialist, please answer each question based on what you would want a PCP to do in order to create a problem list that is most helpful to you.

These scenarios are meant to be answered within the context of working at Partners HealthCare. Therefore, you are using an EHR system with access to the patient's entire medical record. Also every patient's record is shared with other practitioners through the EHR.

8) Donna goes to see her PCP and mentions that she is terrified of getting breast cancer because both her maternal grandmother and mother had breast cancer. Now her sister was recently diagnosed. Should Donna's family history of breast cancer be mentioned on her problem list?
- ☐ Yes
- ☐ No

9) John comes in to a medical center's urgent care ward with a small facial laceration from playing hockey. John mentions he's a male model to the physician and explains that he wants treatment that will minimize scarring. Should the doctor add John's occupation as a model to the problem list?
- ☐ Yes
- ☐ No

10) Tenesha recently moved to Boston and goes to see her new PCP for an annual physical. Tenesha says that she was diagnosed by a pulmonologist with exercise-induced asthma several years ago. Currently, she takes no medications to treat her asthma, is experiencing no symptoms, and the asthma does not affect her daily life. Should 'exercise-induced asthma' be added to her problem list?
- ☐ Yes
- ☐ No

11) Maria is a 52 year old women and is afraid of doctors. She summons up the courage to go see a doctor for the first time in years because of a persistent cough. Should the doctor add a note about Maria's fear of doctors to her problem list?
- ☐ Yes
- ☐ No

12) Jorge appears to have ongoing chest pain, but after a full work up the practitioner can not diagnose the cause. Should the practitioner add an item about chest pain to the Jorge's problem list?
- ☐ Yes
- ☐ No

13) Paul goes to see a psychiatrist and is diagnosed with depression. Should the psychiatrist add 'depression' to Paul's problem list?
- ☐ Yes
- ☐ No

14) Janice goes to see a psychiatrist and is diagnosed with anorexia nervosa. She also goes to see a PCP, allergist, gynecologist, and neurologist at the same medical facility. While her psychiatrist's notes are restricted to the mental health department, all of Janice's other doctors are viewing a common problem list through an electronic health record (EHR) system. Under this scenario, should the psychiatrist add 'anorexia nervosa' to Janice's problem list?
- ☐ Yes
- ☐ No

15) Dr. Thomas works at a mental health facility that encourages psychiatrists to add mental health problems to the patients' problem lists. During one of Dr. Thomas's patient visits, the patient strongly disagrees with the diagnosis of depression. Should Dr. Thomas still list 'depression' on the patient's problem list?

- ☐ Yes
- ☐ No

16) Dr. Brown works at a health center that offers their patients the ability to view their entire electronic health record online through a patient portal. Dr. Brown is with a patient whom he diagnoses with obesity. Dr. Brown knows this patient regularly checks the patient portal to review her medical record. Should Dr. Brown list 'obesity' on this patient's problem list?

- ☐ Yes
- ☐ No

17) Toby appears to have an asthma attack during a soccer game. His PCP refers him to a local pulmonologist. The pulmonologist diagnoses him with asthma and has access to the same electronic health record as the PCP. How should the pulmonologists address the problem list?

- ☐ The pulmonologist should add 'asthma' to Toby's problem list.
- ☐ The pulmonologist should advise the PCP to add 'asthma' to the problem list in his follow up.
- ☐ The pulmonologist should perform his regular feedback and assume the PCP will add 'asthma' to Toby's problem list if the PCP feels it is necessary.
- ☐ Other

18) Ritchie has an appendectomy performed at the local hospital. His PCP gets the medical record from the hospital. Should Ritchie's PCP add 'appendectomy' to Ritchie's problem list?

- ☐ Yes
- ☐ No

19) Paul is hospitalized due to a heart attack caused by his coronary artery disease. At Paul's PCPs office 'coronary artery disease' is already listed on his problem list. Now Paul's PCP receives Paul's medical information from the hospital. Should Paul's PCP add another item specifically mentioning Paul's recent hospitalization to the problem list?

- ☐ Yes
- ☐ No

20) Dr. Baker likes to include long term undiagnosed symptoms on his patients' problem lists. Catherine comes in for her first appointment with Dr. Baker complaining of lower back pain. If Catherine keeps coming to see Dr. Baker once a month complaining of lower back pain, at what appointment/month should Dr. Baker add an item about back pain to Catherine's problem list?

- ☐ 1st appointment/ month
- ☐ 2nd
- ☐ 3rd
- ☐ 4th
- ☐ 5th
- ☐ 6th thru 11th
- ☐ > 12th appointment/ month
- ☐ It depends
- ☐ Never

21) Helen is having her third urinary tract infection (UTI) within one year. Should the practitioner add a statement about the Helen's predisposition for urinary tract infections to her problem list?

- ☐ Yes
- ☐ No

22) The practitioner does mention Helen's predisposition for UTI's on her problem list. Three months later, Helen is in for her annual physical and mentions that she has not had any UTIs for the past three months. Helen continues not to experience anymore UTIs. At what point should the item about Helen's predisposition for UTIs be removed from the problem list?

- ☐ 1-3 months
- ☐ 4-6 months
- ☐ 6-9 months
- ☐ 10-11 months
- ☐ 1-2 years
- ☐ 3-4 years
- ☐ > 5 years
- ☐ Never
- ☐ It depends

23) Sally is diagnosed with Type II diabetes. What term should the practitioner use on Sally's problem list?

- ☐ DM
- ☐ DM II
- ☐ Diabetes Type II
- ☐ Diabetes
- ☐ Diabetes Mellitus
- ☐ Diabetes Mellitus Type II
- ☐ Other

24) Sally develops coronary artery disease as a result of her Type II diabetes. Should the resultant coronary artery disease be listed on the problem list?

- ☐ Yes
- ☐ No

25) If a practitioner wants to list Sally's coronary artery disease, how should coronary artery disease be listed on her problem list?

- ☐ Diabetes Type II with coronary artery disease
- ☐ As a separate problem from Diabetes Type II

26) John recently moved to Boston and is going for his annual physical exam with a new health center. A nurse practitioner is giving John his physical exam and John tells the nurse practitioner that he was diagnosed with asthma by a pulmonologist. Should the nurse practitioner be able to add problems like John's asthma to the problem list or should only physicians be able to add problems?

- ☐ The nurse practitioner should be able to add problems to the problem list.
- ☐ Only physicians should be able to add problems to the problem list.
- ☐ Other

27) Carlos breaks his leg and goes to the hospital. The nurse is performing her medication rounds when Carlos mentions to her that he forgot to tell the triage nurse that he has hemophilia. What should the nurse do in regards to the problem list?

- ☐ The nurse should access Carlos's medical record and add 'hemophilia' to the problem list.
- ☐ The nurse should tell the doctor that Carlos has hemophilia and recommend that the doctor add 'hemophilia' to the problem list.
- ☐ The nurse should tell the doctor that Carlos has hemophilia and assume that Carlos's doctor will add 'hemophilia' to Carlos's problem list without a specific recommendation.
- ☐ Other
