# Supplementary material for: Healthcare provider attitudes towards the problem list in an electronic health record: a mixed-methods qualitative study
Source: BMC Med Inform Decis Mak. 2012 Nov 11;12:127. doi: 10.1186/1472-6947-12-127 (PMC3534408; doi:10.1186/1472-6947-12-127)
Supplement: Additional File 2 — Appendix B. In-Person Responses to the Vignettes. Description of Data: Tabulated data from online questionnaire and relevant quotes from the in-person interviews. [file 1472-6947-12-127-S2.doc]

**APPENDIX A**

**In-Person Interview Responses to Vignettes**

**Contents:** Tabulated data from online questionnaire and quotes from the in-person interviews.

**Note:** The LMR referred to in several of the quotations is the EHR used by the practitioners.

**What problems should be included (broad)?**

| **Question 1: Family History** | **Answers** | **% Res.** | **N** |
| --- | --- | --- | --- |
| Donna goes to see her PCP and mentions that she is terrified of getting breast cancer because both her maternal grandmother and mother had breast cancer. Now her sister was recently diagnosed. Should Donna's family history of breast cancer be mentioned on her problem list? | Yes | 76.24 | 77 |
|  | No | 23.76 | 24 |
|  |  |  |  |
|  |  |  |  |

**Yes Responses:**

*Specialist:* “It should be on the problem list because it might influence the type and nature screening and the discussion of genetic counseling and testing. However, what is put on the problem list is going to be an issue because family history of breast cancer could be my mother had breast cancer. It could be I have 12 relatives with breast cancer. And it could be my mother was 85 when she had breast cancer or my mother was 35 when she had breast cancer. All of those things are completely different. The idea of just saying family history is not useful information unless it qualified.”

*Specialist:* “I would want a family history of breast cancer on her list and I would want to have a note about which of her relatives had it. If it’s her mom's third cousin I don't care, but two first degree relatives and a grandmother, means a lot in terms of the implications for her health. So I would expect that the internist who put that problem on the problem list gets that and made that leap. That's where it’s harder when the nurses are entering it. Because sometimes they will just enter yes, she circles family history on the intact form not like what does it mean in terms of what we do clinically.”

**No Responses:**

*PCP:* “If you want to know the family history, go to the family history section”

*PCP:* “I would say no... There already is a function for family history within LMR and it would seem like you are duplicating things if you’re also putting these things in the problem list.”

| **Question 2: Social History** | **Answers** | **% Res.** | **N** |
| --- | --- | --- | --- |
| John comes in to a medical center's urgent care ward with a small facial laceration from playing hockey. John mentions he's a male model to the physician and explains that he wants treatment that will minimize scarring. Should the doctor add John's occupation as a model to the problem list? | Yes | 7.92 | 8 |
|  | No | 92.08 | 93 |
|  |  |  |  |
|  |  |  |  |

**Yes Responses**

There were no “yes” responses within the in-person interviews.

**No Responses**

*Specialist:* “Should the doctor add John's occupation as a model? No. I wouldn't really want his occupation added to the problem list. That would be labeling. That would be putting a stigma on an occupation. Or qualifies somebody’s occupation which I don't think is helpful.”

*PCP:* “No, I think you know you could put that in social history. You know if he had serious issues with his cosmesis and stuff and you thought he had a body dimorphic disorder, that would be a problem, but his occupation itself is not a problem.”

*Specialist:* “That's a tricky one. Ummm, I would say no that sort of takes some explanation and it’s probably not going to be relevant in any other clinical scenario. It’s hard to think of another scenario that being a model adds to the complexity of his medical care.”

*Interviewer:* Can you think of other occupations you would want to know or is that just too far off?

*Specialist (cont):* “I don't know, I think one of the things we see a lot is health care providers get flagged in their problem list. I think that we shouldn't. We should probably, you know, we should all get the same care, right. I think if there's an occupational exposure that leads to disease where it’s again clinically relevant for ongoing screening. Like works with benzene chronically blah blah blah blah ...removes asbestos. You know something where like there is an ongoing clinical issue that is going to come up again and again where they need repeated screenings and testing. Then I think that is useful.”

| **Question 3: Surgeries** | **Answers** | **% Res.** | **N** |
| --- | --- | --- | --- |
| Ritchie has an appendectomy performed at the local hospital. His PCP gets the medical record from the hospital. Should Ritchie's PCP add 'appendectomy' to Ritchie's problem list? | Yes | 73.47 | 72 |
|  | No | 26.53 | 26 |
|  |  |  |  |

**Yes Responses:**

*Specialist:* “Yes, Ritchie’s PCP should add appendectomy… I think procedures should be on there. As I think as a specialist who does procedures, you like to know what procedures have been done before.”

**No Responses:**

*PCP:* “I think procedures or surgeries should be on the procedure list, not on the problem list. Again it just clutters it up. When you get a patient that has, you know, twelve, thirteen problems on their problem list to be adding family history and procedures and everything else, especially because the problem list doesn’t prioritize, so it just all prints out and you need to search through it to find what you’re looking for.”

*Specialist:* “So this is from my specialty perspective; infectious disease since we see a lot of issues with surgical infections. Umm, I usually incorporate in my patient notes just a surgical list, you know surgeries, it is part of the problem list, but it goes under surgeries and then a list of surgeries they've had. So appendectomy, hysterectomy and the year that it was done. So I find that helpful for me. I can imagine there are plenty of other providers that wouldn’t find that information that useful. So again, I think that’s a….if I were going to have it on the problem list then I would have it as a list just as… it wouldn’t be necessarily appendectomy, it would be surgeries. It would be the problem list and there would be a list of surgeries.”

| **Question 4: Hospitalizations** | **Answers** | **% Res.** | **N** |
| --- | --- | --- | --- |
| Paul is hospitalized due to a heart attack caused by his coronary artery disease. At Paul's PCPs office 'coronary artery disease' is already listed on his problem list. Now Paul's PCP receives Paul's medical information from the hospital. Should Paul's PCP add another item specifically mentioning Paul's recent hospitalization to the problem list? | Yes | 50.00 | 49 |
|  | No | 50.00 | 49 |
|  |  |  |  |
|  |  |  |  |

**Yes Responses:**

*Specialist:* “I would want to see, yes, when they’ve been in the hospital… If they were hospitalized within the past year, I would want to see it on the problem list. Just like an appendectomy, if someone had an appendectomy thirty years ago I don’t really care about it, if they had an appendectomy in the last year I would want to know about it.”

**No Responses:**

*PCP:* “It should go in the comments, the hospitalization isn’t the problem, the coronary artery disease is the problem. So again, otherwise the list gets too long and then you don’t look at it because it’s too long”

**What Problems Should be Included (Detailed)?**

| **Question 5: Latent non-transitive diseases** | **Answers** | **% Res.** | **N** |
| --- | --- | --- | --- |
| Tenesha recently moved to Boston and goes to see her new PCP for an annual physical. Tenesha says that she was diagnosed by a pulmonologist with exercise-induced asthma several years ago. Currently, she takes no medications to treat her asthma, is experiencing no symptoms, and the asthma does not affect her daily life. Should 'exercise-induced asthma' be added to her problem list? | Yes | 82.18 | 83 |
|  | No | 17.82 | 18 |
|  |  |  |  |
|  |  |  |  |
|  |  |  |  |

**Yes Response:**

*Specialist:* “Yes, I think that should be because it could help to clarify in an emergency a clinical finding that might not be possible for the patient to clarify.”

**Maybe Response:**

*Specialist:* “Yeah, that is a grey area, actually. From someone who is… you know… completely well. This is her only issue then I can see why this might make it on to the problem list. Young person. If it’s… you know… you’re going to be adding on to a list of 10 or 15 problems on a chronically ill person where this is not likely to be a big issue for her, then I could see where you wouldn’t put it on the list. The length of a list actually becomes an issue, I think, just like fatigue…attention fatigue.”

*Specialist:* “Tricky, this is one we see a lot so exercise-induced asthma... I would say like if you don't think it’s actively a disease… it would depend on what she looks like. So many people carry that label that don't actual have any clinical relevance. Again it would depend on the clinical impression of how severe it was and how likely it was to occur.”

**No Response:**

*Specialist:* “No, it should be taken off the problem list. The problem list… I mean the reason why I don’t use the problem list, is I don’t know how relevant it is. And if it includes things that have no relevance to me now, if she had… if you had something and that something goes away, is it still a problem and why should I care about it? So I would say no. Problem lists to me is current and otherwise I can look in the past medical history.”

| **Question 6: Non-medical conditions** | **Answers** | **% Res.** | **N** |
| --- | --- | --- | --- |
| Maria is a 52 year old woman and is afraid of doctors. She summons up the courage to go see a doctor for the first time in years because of a persistent cough. Should the doctor add a note about Maria's fear of doctors to her problem list? | Yes | 35.64 | 36 |
|  | No | 64.36 | 65 |
|  |  |  |  |

**Yes Response:**

*PCP:* That one’s a tough one… I actually might put that on the list…hate to say it I might. Because I think, for me in that case it’s something you might want to know upfront as soon as you’re meeting the patient, and you’re not going to know that if you have to search through the chart for it. But I would probably phrase it as a medical problem though like you know… medical anxiety or anxiety of…around…something like that so it more looks like a problem.

**No Response:**

*Nurse Practitioner:* “I don't think this woman having a fear of doctors is all that relevant. She is there now. Probably in the encounter note, I would say ‘without prior medical care as she has a fear of seeing doctors.’ It also gives you a sense of that lady that she is here because this has her worried. This cough… so this picks up extra, shall I say importance.”

| **Question 7: Undiagnosed long term symptoms** | **Answers** | **% Res.** | **N** |
| --- | --- | --- | --- |
| Jorge appears to have ongoing chest pain, but after a full work up the practitioner cannot diagnose the cause. Should the practitioner add an item about chest pain to the Jorge's problem list? | Yes | 96.04 | 97 |
|  | No | 3.96 | 4 |
|  |  |  |  |

**Yes Responses:**

*PCP:* “I think if he has ongoing chest pain of unclear etiology, but you’ve done… you know… all these tests and he still has chest pain, then I would put it on the problem list, because you know he is going to show up somewhere with chest pain, and people need to know that it’s been worked up.”

*Specialist:* “It’s probably worth wild to put in there, but at least so if a practitioner heard him start to complain about chest pain, that he would know that that was not a new finding.”

**No Responses:**

There were no “no” responses within the in-person interviews.

| **Question 8: Multiple occurrences of transitive illness** | **Answers** | **% Res.** | **N** |
| --- | --- | --- | --- |
| Helen is having her third urinary tract infection (UTI) within one year. Should the practitioner add a statement about the Helen's predisposition for urinary tract infections to her problem list? | Yes | 92.86 | 91 |
|  | No | 7.14 | 7 |
|  |  |  |  |

**Yes Responses:**

*PCP:* “Yes, I probably would say yes and I would phrase it as ‘recurrent UTI.’ But that’s me. To let you know that it’s not just one acute…you know I’ll see a patient and they will have strep throat on their problem list. So they had a step throat you know three years ago, or URI, cold. To me it like ok that’s an acute sub illnesses they had, it’s gone. Everyone gets cold. It’s not like that’s… you know… but if their having an issue that is recurring and recurring, like so Helen is having her third UTI in a year so then you know that this is going to be a chronic issue for her. It is helpful to have that on the problem list so ok this is not new. And it might affect the way you treat her actually.”

*Specialist:* “Helen, yes.”

*Interviewer:* “I know some people have very strict definitions that it’s just supposed to be diagnoses…”

*Specialist (cont.)*:“So the PCP’s tend to be anal retentive about this and that is why I stay away from the problem list. I know that they… my definition of a problem list and their definition of a problem list aren’t necessarily the same.”

**No Responses:**

There were no “no” responses within the in-person interviews.

| **Question 9: Sequelae problems** | **Answers** | **% Res.** | **N** |
| --- | --- | --- | --- |
| Sally develops coronary artery disease as a result of her Type II diabetes. Should the resultant coronary artery disease be listed on the problem list? | Yes | 100.00 | 97 |
|  | No | 0 | 0 |
|  |  |  |  |

**Yes Responses:**

*PCP:* “Absolutely, it may be secondary to her sugar control, but it’s a separate problem. The coronary artery disease should absolutely be on the list because you are going to treat that differently then you are going to treat the diabetes.”

*Specialist:* “Yes, I think coronary artery disease should actually be something in the text under the diabetes. But it should also be…I mean like once it’s producing its own procedures and management then yeah.”

**No Responses:**

There were no “no” responses within the in-person interviews.

**Terminology**

| **Question 10: Use of acronyms/ Level of detail of problems** | | |
| --- | --- | --- |
| Sally is diagnosed with Type II diabetes. What term should the practitioner use on Sally's problem list? | | |
| **Answers** | **% Res.** | **N** |
| DM | 1.03 | 1 |
| DM II | 16.49 | 16 |
| Diabetes Type II | 17.53 | 17 |
| Diabetes | 3.09 | 3 |
| Diabetes Mellitus | 1.03 | 1 |
| Diabetes Mellitus Type II | 58.76 | 57 |
| Other | 2.06 | 2 |

**Responses:**

*Specialist:* Either diabetes Type II or Diabetes Mellitus Type II… We use them [acronyms] a lot, but if it’s coded then you might as well use the real thing so there's no confusion.

*PCP:* “Personally, I don’t think it really matters as long as you know she has diabetes…I mean I guess, for me, Type II diabetes would be important to know, but whether its Diabetes Type II, Diabetes Mellitus Type II, or DM II, I would know… I guess I would say no to the DM II because I’m not a fan of abbreviations because that’s clear to me what that means, but that may not clear to other people who may be looking at the chart. And the example I always give is the ophthalmologists because they use all kinds of abbreviations in the notes about the eye exam and you’re like I mean it’s like reading another language and you’re like ok I don’t know anything of what their talking about, and that’s the note they send us and it’s like ok, I can’t interpret that at all. So I would rather have things spelled out, but whether its type II, Diabetes Type II or Diabetes Mellitus Type II, I think anybody would know what that means so I think its fine.”

| **Question 11: Listing of sequelae** | | |
| --- | --- | --- |
| If a practitioner wants to list Sally's coronary artery disease, how should coronary artery disease be listed on her problem list? | | |
| **Answers** | **% Res.** | **N** |
| Diabetes Type II with coronary artery disease | 0.00 | 0 |
| As a separate problem from Diabetes Type II | 100.00 | 97 |

**Yes Responses:**

*Specialist:* “I think you should list it separately from the Type II Diabetes… I think it comes out cleaner and there are things that you are going to do for her coronary artery disease that you might want to add under coronary artery disease that you know, if they have coronary artery disease and you do a stent. Do you do Type II diabetes mellitus with coronary artery disease that was stend’d this last month or do you just have Type II Diabetes and then coronary artery disease post-stent. I like it separate, I think its cleaner.”

**Maybe Response:**

*Specialist:* “I guess I would say it depends on how bad it is. If she has needed three caths and a cabbage, then yeah. You know again, what is it. How much…if it's requiring a dedicated cardiologist to manage her care then like yeah. If it's just like a thing where she is on Lipitor and you know, she’s on Lipitor and an anti-hypertension, and has failed stress test, but isn't being followed closely by cardiology or anything, then maybe it should just go under diabetes.”

**No Responses:**

There were no “no” responses within the in-person interviews.

**When to add or delete problems?**

| **Question 12: Timing (add)** | **Answers** | **% Res.** | **N** |
| --- | --- | --- | --- |
| Dr. Baker likes to include long term undiagnosed symptoms on his patients' problem lists. Catherine comes in for her first appointment with Dr. Baker complaining of lower back pain. If Catherine keeps coming to see Dr. Baker once a month complaining of lower back pain, at what appointment/month should Dr. Baker add an item about back pain to Catherine's problem list? | 1st appointment/month | 27.55 | 27 |
|  | 2nd | 12.24 | 12 |
|  | 3rd | 25.51 | 25 |
|  | 4th | 3.06 | 3 |
|  | 5th | 0.00 | 0 |
|  | 6th thru 11th | 4.08 | 4 |
|  | > 12th | 0.00 | 0 |
|  | It depends | 26.53 | 26 |
|  | Never | 1.02 | 1 |

**Responses:**

*PCP:* “I think if it’s a chronic issue then it can be on the problem list. Like chronic low back pain to me is a problem. I guess…there’s not a right answer to this one. For me, you know, if it’s chronic then it’s at least 3 months or more. So, I don’t know, the third appointment or fourth appointment for the same issue. Third, I would probably say the third.”

*PCP:* “Probably add it to the list at the second visit…yeah…I think there’s a lot of people who come with symptoms that are transitive and you spend half an hour discussing them one time and the next time they come in they have forgotten all about it and I’m not sure that necessarily deserves a problem on the list, but if someone has a system and continues that problem the next time you see them, you should add it as opposed to these self-limited transient illnesses.”

| **Question 13: Timing (delete)** | **Answers** | **% Res.** | **N** |
| --- | --- | --- | --- |
| The practitioner does mention Helen's predisposition for UTI's on her problem list. Three months later, Helen is in for her annual physical and mentions that she has not had any UTIs for the past three months. Helen continues not to experience any more UTIs. At what point should the item about Helen's predisposition for UTIs be removed from the problem list? | 1-3 months | 6.12 | 6 |
|  | 4-6 months | 4.08 | 4 |
|  | 6-9 months | 5.10 | 5 |
|  | 10-11 months | 4.08 | 4 |
|  | 1-2 years | 38.78 | 38 |
|  | 3-4 years | 6.12 | 6 |
|  | >5 years | 2.04 | 2 |
|  | Never | 8.16 | 8 |
|  | It Depends | 25.51 | 25 |

**Responses**

*Nurse Practitioner:* “And then the next question follows up. At what point should the UTIs be removed from the problem list? I would say it depends, three in one year requires a workup and then I would substitute the recurrent urinary tract infections with whatever the findings are.”

*Specialist:* “Yes, here is an example of how to get rid of things. Yeah, I would say probably 1 to 2 years for this problem, each ones different though. Another example is people with recurrent episodes of cellulitis and you know people can go a couple years without symptoms so you would have to move it to 3-4 years or maybe even more than 5 years, so its diagnosis dependent.”

*PCP:* “That’s a little bit of a dilemma. I would say one to two years… Again, this is different than something like…I’ve had patients who were diagnosed as diabetic actually lose weight and so their diabetes is actually quote “cured”. But I don’t take it off the problem list because I know if they gain weight again then I know they are going to be diabetic again. So, but this is kind of again more of an acute illnesses not a chronic illnesses. So I guess if she goes for a prolonged period without any issues… I’m trying to think do I actually take it off.. sometimes I just put history of instead. And then if it’s been a long long time then I’ll take it off. Maybe one to two years isn’t the right answer, because I probably don’t take it off after a year.”

**Sensitive Problems**

| **Question 14: Whether to include sensitive problems?** | **Answers** | **% Res.** | **N** |
| --- | --- | --- | --- |
| Paul goes to see a psychiatrist and is diagnosed with depression. Should the psychiatrist add 'depression' to Paul's problem list? | Yes | 98.98 | 97 |
|  | No | 1.02 | 1 |

**Yes Responses:**

*PCP:* “Absolutely it should be on there… I know some people don’t want to put any mental health issues on the problem list, but I think its important information that all providers need to know about.”

**No Responses:**

There were no “no” responses within the in-person interviews.

| **Question 15: To include sensitive problems when other practitioners have access to the same record** | **Answers** | **% Res.** | **N** |
| --- | --- | --- | --- |
| Janice goes to see a psychiatrist and is diagnosed with anorexia nervosa. She also goes to see a PCP, allergist, gynecologist, and neurologist at the same medical facility. While her psychiatrist's notes are restricted to the mental health department, all of Janice's other doctors are viewing a common problem list through an electronic health record (EHR) system. Under this scenario, should the psychiatrist add 'anorexia nervosa' to Janice's problem list? | Yes | 98.98 | 97 |
|  | No | 1.02 | 1 |
|  |  |  |  |
|  |  |  |  |
|  |  |  |  |

**Yes Responses:**

*Nurse Practitioner:* “If someone is taking care of the patient, they need to know what the patient history is. Now if she went with a sprained ankle to an orthopedic doctor they might see depression or just keep moving on. But I wouldn't mind that they saw that.”

*PCP:* “Yes, so yeah…same reasons and I think especially in that diagnosis where there are medical complications for the other providers to be unaware to even know that they should be looking for hypoglycemia or cardiac conduction abnormalities or whatever related to her anorexia, I mean that would be….so absolutely.”

*Specialist*: “I don't know what the rules are under this, but I think the diagnosis is relevant to everybody else. So the question is I don't know what the legality of like mental health records is and how visible they are is, but that is where I would defer to someone and say I don't know. If there's a way where it’s not illegal to disclose that, then absolutely.”

**Maybe Response:**

*Specialist:* “I mean on the one hand, I think it encourages or continues the stigma of saying we shouldn't let people know about these things because they’re so private. But on the other hand, so in that sense I think it could actually lessen the stigma by being more commonly known. I certainly understand the legitimate reasons why privacy is important. So that would have to be… I guess I would answer that in the ideal world it would be good for healthy medical care for all clinicians to know all problems about their patients. But I guess I could certainly live with a situation where it was left off the list and accessible by some other mechanism. In other words, it doesn't need to be on a public billboard.”

**No Responses:**

There were no “no” responses within the in-person interviews.

| **Question 16: To include sensitive problems when a patient disagrees with a diagnosis** | **Answers** | **% Res.** | **N** |
| --- | --- | --- | --- |
| Dr. Thomas works at a mental health facility that encourages psychiatrists to add mental health problems to the patients' problem lists. During one of Dr. Thomas's patient visits, the patient strongly disagrees with the diagnosis of depression. Should Dr. Thomas still list 'depression' on the patient's problem list? | Yes | 75.51 | 74 |
|  | No | 24.49 | 24 |
|  |  |  |  |
|  |  |  |  |
|  |  |  |  |

**Yes Responses:**

*PCP :* “Yes, you can always put a qualifier that the patient disagrees. We’ve come across that with things like drug use or alcohol or other issues. Do you put that on the problem list? Patients get upset if they see it. But again I think if it’s important information for other providers to be aware of then I think it has to go on. Then… you know… you can always qualify something to say patient disagrees with the diagnosis. But if they are clinically depressed then they are clinically depressed whether the patient wants to be or not."

**Maybe Response:**

*Specialist:* “Well, this is again tough. I mean it's hard because mental health is the only place where we defer… we allow that people can disagree on diagnosis. I don't know, even though we disagree on diagnosis all the time in other places, we wouldn't say the patient doesn't agree that they have asthma so they don't have asthma. Umm, I think it would come down to what the patient has said can be disclosed to other providers.”

**No Responses**

*Specialist:* “I would not. I would say this is a patient… this is a situation where records are public and will be distributed to other physicians and unless there is sort of an agreement on the listing of the problems or the way that they are described, I would say they probably shouldn’t be on the problem list.”

| **Question 17: To include sensitive problems when a patient has access to the problem list through an online patient portal** | **Answers** | **% Res.** | **N** |
| --- | --- | --- | --- |
| Dr. Brown works at a health center that offers their patients the ability to view their entire electronic health record online through a patient portal. Dr. Brown is with a patient whom he diagnoses with obesity. Dr. Brown knows this patient regularly checks the patient portal to review her medical record. Should Dr. Brown list 'obesity' on this patient's problem list? | Yes | 93.94 | 93 |
|  | No | 6.06 | 6 |
|  |  |  |  |
|  |  |  |  |
|  |  |  |  |

**Yes Responses:**

*Specialist:* “Yeah, so I would say it in a different way. If I was going to put it in, I would say “excessive BMI” or “Body Mass Index is excessive.” I think the words are important but there are other ways to say the same thing without using inflammatory words, I mean people might react to obesity one way and they might react to elevation or excess of body mass index in a totally different way. It’s a less inflammatory way of saying you need to lose weight.”

*PCP:* “Yes, I mean the patient may not like the fact that their obese, but their obese, then the question is maybe it motivates them to do something. You know, hopefully Dr. Brown has discussed this with the patient already that you know your weight is an issue, you meet the criteria for obesity, we need to lose weight, etc. So it shouldn’t be a surprise when the patient sees it.”

*Specialist:* “Absolutely, it’s one of the reasons why I think sharing medical records is a bad idea. But what can you do?...Because what it’s going to do is its going to change what people write in the record and I think that's bad.”

**No Responses:**

There were no “no” responses within the in-person interviews.

**Who can change the problem list across the following roles:**

| **Question 18: Specialist/ PCP** | | |
| --- | --- | --- |
| Toby appears to have an asthma attack during a soccer game. His PCP refers him to a local pulmonologist. The pulmonologist diagnoses him with asthma and has access to the same electronic health record as the PCP. How should the pulmonologists address the problem list? | | |
|
|
| **Answers** | **% Res.** | **N** |
| The pulmonologist should add 'asthma' to Toby's problem list. | 77.78 | 77 |
| The pulmonologist should advise the PCP to add 'asthma' to the problem list in his follow up. | 9.09 | 9 |
| The pulmonologist should perform his regular feedback and assume the PCP will add 'asthma' to Toby's problem list if the PCP feels it is necessary. | 12.12 | 12 |
| Other | 1.01 | 1 |

**Responses:**

*Specialist:* “Oh yes, this is I would say the crux of the issue of the problem list. Who has ownership? That is really, really challenging. Yeah, I don’t know the answer to that actually. I think…you know… one model would be that if you go to see someone with a special expertise for a problem and they feel like you have that problem then either the specialist or primary care provider could put it on the problem list. I think more of an issue is how things come off because otherwise you end up generating these really long lists and if the problem is no longer active and you are not seeing the specialty person anymore then it just stays on there, because often the primary care provider doesn’t feel comfortable taking it away. So I think someone has to have a sort of “keeping the list in perspective role” and that might be the primary care doctor who takes things off the list, if the list is getting too long and the patient is actively being seen by them.”

*Specialist:* “The pulmonologist should in an ideal world add asthma. But it’s not an ideal world. I let the PCP. I think of the problem list as not my list. So I always feel like I’m impinging on the PCP.”

*PCP:* “So specialist should add asthma to the problem list, yes… yeah, I think they have as much responsibility to keep the chart up to date as we do.”

| **Question 19: Nurse practitioner** | | |
| --- | --- | --- |
| John recently moved to Boston and is going for his annual physical exam with a new health center. A nurse practitioner is giving John his physical exam and John tells the nurse practitioner that he was diagnosed with asthma by a pulmonologist. Should the nurse practitioner be able to add problems like John's asthma to the problem list or should only physicians be able to add problems? | | |
|
|
| **Answers** | **% Res.** | **N** |
| The nurse practitioner should be able to add problems to the problem list. | 93.81 | 91 |
| Only physicians should be able to add problems to the problem list. | 6.19 | 6 |
| Other | 0.00 | 0 |

**Responses:**

*Specialist:* “Well, I mean nurse practitioners see a lot of patients and provide primary care so I don’t think you could take away….if you say they can’t add to the problem list then the problem lists are going to be incomplete. I think its fine, but it’s going to be an education issue. But I mean there's no reason they will be any different at it than any other health care provider.”

*PCP:* “No issue. Definitely not. We work very closely with nurse practitioners down at the clinic and they see a lot of our patients so we absolutely rely on them”

| **Question 20: Other RN** | | |
| --- | --- | --- |
| Carlos breaks his leg and goes to the hospital. The nurse is performing her medication rounds when Carlos mentions to her that he forgot to tell the triage nurse that he has hemophilia. What should the nurse do in regards to the problem list? | | |
|
|
| **Answers** | **% Res.** | **N** |
| The nurse should access Carlos's medical record and add 'hemophilia' to the problem list. | 22.68 | 22 |
| The nurse should tell the doctor that Carols has hemophilia and recommend that the doctor add 'hemophilia' to the problem list. | 60.82 | 59 |
| The nurse should tell the doctor that Carols has hemophilia and assume that Carlos's doctor will add 'hemophilia' to Carlos's problem list without specific recommendation. | 14.43 | 14 |
| Other | 2.06 | 2 |

**Responses:**

*Specialist:* Nurse medication rounds…yeah, this one’s harder. I mean for this particular example, it’s such a serious problem that… you know… that yes it should be on his problem list and in this particular case, the nurse could put it on the problem list and it’s probably fine. On the other hand what the triage nurses hear every day is thousands of things that might possible make it on the problem list. I think that probably is not what you want to have happen. So if the question is can nurses add on to the problem list, I would think – this is going to get me into trouble – but probably not. Are we going miss a few things? Yes definitely, but will the balance still be favorable, yes, I think so. Nurse practitioners are an exception because their providing primary care. Nurses providing primary care should be able to. “

*PCP:* “So this is, I wouldn’t have the nurses add it without the doctors, because again the doctor hasn’t made the diagnosis or the provider, it’s just a piece of history from the patient, so I would have probably two, the nurse should make the doctor aware of this new diagnosis and recommend that they add it to the problem list. You know, then if the doctor says well why don’t you go ahead and add it to the problems, then that would be fine to. But I guess I would want someone signing off on that before the nurses went ahead and did it.”

*PCP:* “I guess my experience has been that… you know… both doctors and nurses are not updating those LMR problem lists at the hospital so I would not expect anyone to change it. I've never seen people amending the problem list on an inpatient, apart from maybe their PCP. If their PCP works in a practice in which they use LMR and then one of their clinic patients is hospitalized with a new medical problem they may add it. If it’s just residents and nurses, I haven't seen that being done.”
